# Knowledge, attitude and practice of Nigerian women towards breast cancer: A cross-sectional study

**DOI:** 10.1186/1477-7819-4-11

**Published:** 2006-02-21

**Authors:** Michael N Okobia, Clareann H Bunker, Friday E Okonofua, Usifo Osime

**Affiliations:** 1 Department of Epidemiology, Graduate School of Public Health, University of Pittsburgh, Pittsburgh, PA 15261, USA; 2 Department of Surgery, College of Medical Sciences, University of Benin, Benin City, Nigeria; 3 Department of Obstetrics and Gynecology, College of Medical Sciences, University of Benin, Benin City, Nigeria

## Abstract

**Background:**

Late presentation of patients at advanced stages when little or no benefit can be derived from any form of therapy is the hallmark of breast cancer in Nigerian women. Recent global cancer statistics indicate rising global incidence of breast cancer and the increase is occurring at a faster rate in populations of the developing countries that hitherto enjoyed low incidence of the disease. Worried by this prevailing situation and with recent data suggesting that health behavior may be influenced by level of awareness about breast cancer, a cross-sectional study was designed to assess the knowledge, attitude and practices of community-dwelling women in Nigeria towards breast cancer.

**Methods:**

One thousand community-dwelling women from a semi-urban neighborhood in Nigeria were recruited for the study in January and February 2000 using interviewer-administered questionnaires designed to elicit sociodemographic information and knowledge, attitude and practices of these women towards breast cancer. Data analysis was carried out using Statistical Analysis Software (SAS) version 8.2.

**Results:**

Study participants had poor knowledge of breast cancer. Mean knowledge score was 42.3% and only 214 participants (21.4%) knew that breast cancer presents commonly as a painless breast lump. Practice of breast self examination (BSE) was low; only 432 participants (43.2%) admitted to carrying out the procedure in the past year. Only 91 study participants (9.1%) had clinical breast examination (CBE) in the past year. Women with higher level of education (X^2 ^= 80.66, p < 0.0001) and those employed in professional jobs (X^2 ^= 47.11, p < 0.0001) were significantly more knowledgeable about breast cancer. Participants with higher level of education were 3.6 times more likely to practice BSE (Odds ratio [OR] = 3.56, 95% Confidence interval [CI] 2.58–4.92).

**Conclusion:**

The results of this study suggest that community-dwelling women in Nigeria have poor knowledge of breast cancer and minority practice BSE and CBE. In addition, education appears to be the major determinant of level of knowledge and health behavior among the study participants. We recommend the establishment and sustenance of institutional framework and policy guidelines that will enhance adequate and urgent dissemination of information about breast cancer to all women in Nigeria.

## Background

Breast cancer is the most common cancer and the second principal cause of cancer deaths in women worldwide as well as in Nigeria [1,2]. The incidence of the disease appears to be rising faster in population groups that have hitherto enjoyed low incidence. The peak age of breast cancer in Nigerian women is about a decade earlier than Caucasians [3-5]. For women with symptomatic breast cancer, prolonged delay, defined arbitrarily as an interval greater than 3 months from first detection to time of diagnosis and treatment has been shown to be associated with increased tumor size [6,7] and more advanced stage of disease [7,8] and with poor long-term survival [6,9]. An estimated 20–30% of Caucasian women wait for at least 3 months before seeking help for breast cancer symptoms [10] compared with over 70% of Nigerian women presenting with advanced stages at which time little or no benefit is derived from any form of therapy [3-5]; the 5-year survival of breast cancer in Nigeria is less than 10% [3] compared with over 70% in Western Europe and North America. The recent fall in deaths from breast cancer in Western Nations is partly explained by earlier diagnosis as a result of early presentation. Understanding the factors that influence patient delay is a prerequisite for strategies to shorten delays [11].

Although there is strong evidence suggesting that older women in the developed countries are more likely to delay their presentation with breast cancer, [12], there is data suggesting that factors related to women's knowledge and beliefs about breast cancer and its management may contribute significantly to medical help-seeking behaviors [13-15]. The three screening methods recommended for breast cancer includes breast self-examination (BSE), clinical breast examination (CBE), and mammography. Unlike CBE and mammography, which require hospital visit and specialized equipments and expertise, BSE is inexpensive and is carried out by women themselves. Several studies, based on breast cancer patient's retrospective self-report on their practices of the exam, have established that a positive association exists between performance of the exam and early detection of breast cancer [16]. There is also evidence that most of the early breast tumors are self-discovered [17] and that the majority of early self-discoveries are by BSE performers [17].

Breast cancer presents most commonly as a painless breast lump and a smaller proportion with non-lump symptoms. For women to present early to hospital they need to be "breast aware"; they must be able to recognize symptoms of breast cancer through routine practice of practicable screening. At the present time, routine mammography cannot be recommended in developing countries due to financial constraints and the lack of accurate data on the burden of breast cancer in these countries. Few studies have examined the knowledge, attitude and practice of women towards breast cancer in Nigeria [13,18,19]. These studies are often of small sample size and targeted women in special professions. We are unaware of any study that has examined these issues in community-dwelling women who constitute the majority of at risk women both for the disease and late presentation. This study recruiting 1000 community-dwelling women from an urban community in Nigeria was designed to evaluate the knowledge, attitude, and practice of these women towards breast cancer.

## Methods

The study was designed to assess knowledge, attitude and practice towards breast cancer among 1000 community-dwelling women recruited from Egor local government area, a semi-urban community with a population of 229,681 comprising 115,550 males and 114,131 females in Edo State of Nigeria (1991 Population Census of Nigeria). Participants were recruited from randomly selected households using the 1991 National Population Census database listing of households in the local government area. For participants that declined participation, other households were randomly selected until the sample size of 1000 participants was achieved. University of Benin Research and Ethics Committee approved the study protocol and written informed consent was obtained from study participants prior to recruitment. Participants were recruited in their homes in January and February 2000.

Data collection was accomplished using interviewer-administered questionnaires designed to obtain relevant socio-demographic characteristics, knowledge, attitude and practice towards breast cancer. The questionnaire was developed by the authors based on information in the literature on risk-factors, common symptoms and signs of breast cancer, common methods of early detection, and current treatment modalities for the disease. The questionnaire was reviewed by a senior oncologist in our institution, who is not among the authors. The questionnaire was pre-tested on a convenient sample of 25 women drawn from the local community from whom study participants were recruited. As a result of this pretest, some of the items on the questionnaire were discarded mostly due to ambiguity of these questions. Some other questions were revised to ensure that vocabulary was within the comprehension of study participants while at the same time retaining the message of the question. Twenty core questions on knowledge of study participants on risk factors, common symptoms, methods of early detection, and treatment methods of breast cancer were retained out of the initial 31 items on the questionnaire. Except for the section of socio-demographic characteristics, most of the questions were designed to elicit "yes", "no" or "don't know" answers.

Participants were recruited by trained nurses, who had two days training session prior to commencement of study. Each respondent was identified by a study identification number. Socio-demographic information relating to age, educational status, religion, occupation, and marital status were collected in the first section of the questionnaire. In the second part, respondents were asked specific questions to elicit their knowledge of the common symptoms and signs of breast cancer, etiological factors, diagnostic procedures, and treatment options available for the disease. The third section examined participant's action and attitude towards breast cancer. Respondents were asked questions related to their practice of breast self examination (BSE), clinical breast examination (CBE) and mammography screening. Participants were also asked about specific actions they will take in the event of being diagnosed with breast cancer and acceptance of mastectomy as a treatment procedure.

Data analysis was carried using the Statistical Analysis Software (SAS) Version 8.2. Each respondent was scored based on the number of correct answers on 20 questions related to knowledge of common symptoms and signs of breast cancer, causal factors and available treatment options and percentage scores were computed. Pearson chi-square test was used to assess relationship between percentage scores and sociodemographic variables. We examined the practice of breast self examination (BSE) to determine factors that might influence this behavior. Variables considered for this analysis included education, age, marital status, and religion. Unconditional logistic regression models were used to assess the relationship of these variables with practice of BSE. Dummy variables were created for nominal categorical variables prior to the logistic regression. Univariate unconditional logistic regression models were first used to assess the relationship of these variables with practice of BSE; those with p > 0.25 were then dropped. Next, variables with p ≤ 0.25 were selectively added to the model, starting with the variable with the lowest p-value. Variables with p ≤ 0.10 were retained while those with p > 0.10 were dropped from the model. At the next stage variables with p > 0.05 were selectively removed from the model, one at time, beginning with the variable with the highest p-value. The model was then re-assessed, followed by dropping of the variable with the next highest p-value until all the variables with p > 0.05 were eliminated from the final multivariate model model. In addition, relationship between these variables and acceptance of mastectomy was similarly examined using unconditional logistic regression.

## Results

One thousand community-dwelling women out of a total of 1052 potential participants that were contacted for the study were recruited from a semi-urban local government area in Nigeria.

### Sociodemographic characteristics of study participants

The sociodemographic characteristics of study participants are shown in Table 1. Of the 1000 community-dwelling women that participated in this study, information about age was available on 826 respondents. Their ages ranged from 15–91 years with a mean of 29.13 ± 10.50 years. About half of the study participants were married. Majority were Christians while forty-five participants (4.7%) were Muslims. About 24.4% of the participants had primary education, 48.7% had secondary education, 12.4% had National Certificate of Education (NCE)/Polytechnic education while 11.9% had University education.

**Table 1:** Socio-demographic characteristics of study participants

**Age distribution**	**Number**	**Percentage**
***Under 30***	512	61.9
***30–39***	192	23.2
***40–49***	82	9.9
***50 or older***	40	4.8
		
Marital status		
***Married***	484	49.4
***Single***	451	46.1
***Divorced/separated***	21	2.1
***Widowed***	23	2.3
		
Religion		
***Christainity***	817	85.0
*Catholic*	271	
*Prostestant*	54	
*Pentecostal*	492	
***Islam***	45	4.7
***Others***	99	10.3
		
Education		
***Primary school***	222	24.4
***Secondary school***	442	48.7
***NCE/Polytechnic***	113	12.4
***University***	108	11.9
***Others***	23	2.5

#### Knowledge about breast cancer

Twenty questions with "yes", "no", or "don't know" responses were designed to elicit participant's knowledge in three key areas, including risk factors for breast cancer and common symptoms, methods of early detection and diagnosis, and their attitudes and practices including practice of breast self examination (BSE), clinical breast examination (CBE) and mammography screening. Table 2 shows participant's responses to some selected questions.

**Table 2:** Response of participants to selected questions on breast cancer

**Knowledge**	**Number**	**Percentage**
Breast cancer is the most common cancer in women		
Yes	673	67.3
No	235	23.5
I don't know	192	19.2
		
Breast cancer occur more commonly in old people		
Yes	254	25.4
No	626	62.6
I don't know	120	12.0
		
Breast can be inherited		
Yes	244	24.4
No	567	56.7
I don't know	189	18.9
		
Breast cancer is caused by evil spirits		
Yes	400	40.0
No	455	45.5
I don't know	145	14.5
		
Breast cancer usually present as a painless breast lump		
Yes	214	21.4
No	521	52.1
I don't know	265	26.5
		
Early diagnosis improves outcome of treatment		
Yes	825	82.5
No	65	6.5
I don't know	110	11.0
		
Breast self examination is useful in early diagnosis		
Yes	872	87.2
No	19	1.9
I don't know	109	10.9
		
Breast cancer is curable when detected early		
Yes	414	41.4
No	460	46.0
I don't know	126	12.6

Study participants had good knowledge of the burden of breast cancer in women. A large proportion of participants (673 [67.3%]) agreed that breast cancer is a major problem in women and 726 participants (72.6%) correctly identified breast cancer as the most common cancer in women. Knowledge of study participants about risk factors for breast cancer was low. For example, in response to the question on the inheritability of breast cancer, only 244 (26.2%) were aware that breast cancer could be inherited in some families. There were many erroneous impressions about the etiology of breast cancer. A large proportion of participants (400 [40.0%]) believed that evil spirits causes breast cancer and 259 (25.9%) indicated that breast cancer results from an infection.

Participant's knowledge about symptoms of breast cancer was rather poor. Only 214 participants (21.4%) knew that breast cancer presents commonly as a painless breast lump. Fewer participants were able to respond correctly to questions on non-lump symptoms of breast cancer such as pain in the breast, nipple discharge, and ulceration of the nipple. In terms of methods of diagnosis, only 432 participants (43.2%) were able to correctly identify breast self-examination (BSE) as a method for detection of breast cancer. A very small proportion of study participants indicated mammography as enhancing in early detection of breast cancer. Four hundred and fourteen participants (41.4%) correctly noted that breast cancer is curable when detected early.

#### Attitude towards breast cancer

There was an indication of positive medical help-seeking behavior as majority of participants indicated visiting the doctor as the best approach to breast cancer care. Only 82 (8.2%) indicated visiting alternative health practitioners for breast cancer care. In terms of practice, only 349 participants (34.9%) practice BSE; the source of information about BSE was from the doctors' offices in 91 participants (21.1%), leaflets in 117 (27.1%), televisions in 134 (31.0%), churches/religious organizations in 35 (8.1%), feminist organizations in 29 participants (6.7%) and Nigerian Cancer Society programs in 26 participants (6.0%). Only 91 participants (9.1%) had clinical breast examination (CBE) in the past year. The main reasons advanced for not having clinical breast examination (CBE) include not having a breast problem in majority of the participants (568, 62.5%) and being unaware of the need for CBE in 293 participants (32.2%) as shown in Table 3. None of the participants has ever had mammography screening.

**Table 3:** Distribution of respondents according to practice of breast self examination (BSE) and clinical breast examination (CBE)

**Practice of breast self examination**	**Number**	**Percentage**
Yes	349	34.9
No	651	65.1
		
Frequency of practice of breast self examination		
Once a month	244	69.9
Once in two months	15	4.3
Three to five times a year	82	23.5
Once or twice a year	8	2.3
		
Source of knowledge of breast self examination		
From a doctor	91	21.1
Publications	117	27.1
Television	134	31.0
Churches/religious groups	35	8.1
Women organizations	29	6.7
Nigerian Cancer Society programs	26	6.0
		
Reasons for not practicing breast self examination		
I don't have breast problem	328	50.4
I don't think I should	154	23.7
I just don't feel like doing it	45	6.9
I don't think I will find anything	22	3.4
I leave it for doctors and nurses to do	18	2.8
Carelessness	14	2.2
I am not pregnant	12	1.8
Laziness	4	0.6
I don't know	54	8.3
		
Practice of clinical breast examination	Number	Percentage
Yes	91	9.1
No	909	90.9
		
Reasons for not practicing clinical breast examination		
I don't have breast problem	568	62.5
I didn't know that I should	293	32.2
I don't know	48	5.3

#### Determinants of breast knowledge score

We computed percentage score of participants' knowledge of breast cancer in order to examine the determinants of breast cancer knowledge. The mean score of the participants was rather low (42.3% ± 12.3). Only 229 participants (22.9%) scored 50.0% and above. Performance was found to be significantly related to level of education and occupation as shown in Table 4. Among 739 participants with complete information on education and knowledge scores, we found that majority of the participants with primary school education (163 [84.9%]) scored below 50.0%. Two hundred and eighty-one participants (76.6%) with secondary education had scores below 50%. Of those with NCE/Polytechnic education, 47.3% scored below 50.0% while 43.8% of those with University education had scores below 50.0%. Chi square test showed a significant relationship between education and level of performance (X^2 ^= 80.66, p < 0.0001). Participants engaged in self-employed small businesses such as trading and hair dressing and secretarial jobs had significantly poorer scores compared with those employed in professional jobs such as sales, teaching, and nursing (X^2 ^= 47.11, p < 0.0001). Although age was not significantly related to scores, we found that older women appear to have higher scores compared with younger women. Forty percent of women aged 50 years and above compared with 35.5% of those aged 40–49 years and 28.4% of those below the age of 40 years scored 50.0% and above (X^2 ^= 3.23, p = 0.12). Religion was not significantly related to scores as shown in Table 4.

**Table 4:** Distribution of knowledge of breast cancer according to sociodemographic variables

**Variables**	**Scores in percentage**			
	<50.0	≥ 50.0	X^2^	P-value
**Education**			80.6	P < 0.0001
***Primary***	163 (84.9)	29 (15.1)		
***Secondary***	281 (76.6)	86 (23.4)		
***NCE/Polytechnic***	43 (47.3)	48 (52.7)		
***University***	39 (43.8)	50 (56.2)		
				
**Occupation**			47.1	P < 0.0001
***Trading***	155 (65.4)	82 (34.6)		
***Business***	230 (76.7)	70 (23.3)		
***Hairdressing***	130 (79.8)	33 (20.2)		
***Profesionals (teaching, nursing, sales)***	14 (33.3)	28 (66.7)		
				
**Age**			3.20	P = 0.199
***< 40 years***	396 (71.6)	157 (28.4)		
***40–49 years***	40 (64.5)	22 (35.5)		
***≥ 50 years***	21 (60.0)	14 (40.0)		
				
**Religion**			3.32	P = 0.506
Catholic	144 (67.6)	69 (32.4)		
***Anglican***	29 (60.0)	13 (31.0)		
***Pentecostal***	297 (74.1)	104 (25.9)		
***Muslim***	28 (75.7)	9 (24.3)		
***Others***	58 (72.5)	22 (27.5)		

#### Determinants of breast self examination

Eight hundred and sixty-six participants had information on education and practice of BSE. A smaller proportion (31.8% [209]) of study participants with high school education and below practiced BSE compared with 62.3% (132) of those with education above high school. Higher level of education was significantly associated with practice of BSE (Odds ratio [OR] = 1.61, 95% Confidence Interval [CI] 1.42–1.88).

Next, we assessed the association of knowledge score with practice of BSE. There were 791 study participants with complete information on knowledge scores and practice of BSE. One hundred and eighty (30.9%) participants with knowledge scores below 50.0% practiced BSE compared with 131 participants (58.0%) of those with knowledge scores of 50.0% and above. Unconditional logistic regression showed a significant association between knowledge scores and practice of BSE. Participants with higher knowledge scores were about 3 times more likely to practice BSE compared with those with scores below 50.0% (OR = 2.95, 95% CI 2.15–4.05). In the final unconditional logistic regression models, only education (OR = 1.53, 95% CI 1.31–1.80) and knowledge scores (OR = 2.40, 95% CI 1.71–3.38) remained in the model. There was no significant association between age, religion and marital status and practice of BSE as shown in Table 5.

**Table 5:** Association of practice of BSE and acceptance of Mastectomy with relevant variables

**Variable**	**Practice of BSE**		**Odds ratio (OR)**	**95% Confidence Interval (CI)**
	Yes	No		
Education				
> High School	132 (62.3)	80 (37.7)	3.56	2.58–4.92
≤ High School	209 (31.7)	451 (68.3)	1.00	
				
Age				
≥ 50 years	14 (46.7)	16 (53.3)	1.41	0.68–2.93
< 50 years	274 (38.3)	441 (61.7)	1.00	
				
Knowledge score				
≥ 50.0%	131 (58.0)	95 (42.0)	2.95	2.15–4.05
< 50.0%	180 (31.9)	385 (68.1)	1.00	
				
	Acceptance of Mastectomy			
Education				
> High School	133 (65.8)	69 (34.2)	3.56	2.58–4.92
≤ High School	340 (51.2)	324 (48.8)		
				
Age				
≥ 50 years	24 (70.6)	10 (29.4)	1.41	0.68–2.93
< 50.0%	375 (53.4)	327 (46.6)	1.00	
				
Knowledge score				
≥ 50.0%	151 (69.9)	284 (49.7)	2.95	2.15–4.05
< 50.0%	65 (30.1)	287 (50.26)	1.00	

#### Determinants of mastectomy acceptance

The relationship between acceptance of mastectomy and sociodemographic variables and knowledge score were also assessed using unconditional logistic regression. Eight hundred and sixty-six participants had complete information on education and acceptance of mastectomy as a treatment of modality. Fifty-one percent (340) of study participants with high school education and below indicated accepting mastectomy compared to 65.8% (133) of those with education above high school. Participants with higher level of education were 1.3 times more likely to accept this treatment procedure compared with those with high school education and below (OR = 1.31, 95% CI 1.14–1.50). As shown in Table 5, 69.9% (151) of participants with higher knowledge scores of 50.0% and above indicated accepting mastectomy compared with 49.7% (284) of those with knowledge scores below 50.0%. Women with higher knowledge scores were 2.36 times more likely to accept mastectomy compared to those with lower knowledge scores (OR = 2.36, 95% CI 1.69–3.30). Age, marital status and religion were not significantly associated with acceptance of mastectomy. In the final unconditional logistic regression model, both education (OR = 1.18, 95% CI 1.01–1.38) and high knowledge scores (OR = 2.15, 95% CI 1.52–3.06) remained as significant predictors of mastectomy acceptance.

## Discussion

The results of this study suggest that community-dwelling women in Nigeria have rather poor knowledge of breast cancer. This may partly explain the late presentation seen in over 70% of women with the disease [3-5]. A mean knowledge score of 42.3% with only 22.9% scoring 50.0% and above portray the abysmal level of ignorance about risk factors and common symptoms of breast cancer in Nigerian women. Unlike previous studies on this subject in Nigerian women [13,18,19], we have recruited community-dwelling women spanning a wide spectrum of age, occupation and educational status. The wide age coverage was deliberate as breast cancer shows a younger age profile in Nigerian women similar to reports in other populations of black descent in the Diaspora but contrary to the older age distribution in Caucasian women; the reported mean ages of 38, 44, and 48 years at presentation reported by various investigators [3-5] in Nigeria support this proposition.

The low level of knowledge found in this study is in keeping with reports of other investigators [13,18,19]. In a survey of breast cancer knowledge, Uche [18] noted that only 32% of the respondents knew that a breast lump was a warning sign for breast cancer, 58.5% were unaware of most warning signs and only 9.8% knew of methods of detecting breast cancer. Our study showed that only 21.4% of community-dwelling women were aware of a painless breast lump as a common presentation of breast cancer and far less proportion of these women were able to identify non-lump presenting symptoms of breast cancer, while only 43.2% were aware of BSE as a screening tool for breast cancer. Even professional health workers such as nurses who are supposed to be leaders in "breast awareness", were reported to have similar low knowledge scores [13]. Odusanya and Tayo [13] found that only 27% of nurses in a tertiary health institution in Lagos, Nigeria could identify up to 3–4 risk factors for breast cancer. In addition, 51% of these nurses wrongly identified the use of fingertips in performing BSE.

These results in Nigerian women sharply contrast with reports from the Western world. In a study of women's knowledge and belief about breast cancer among British women, Grunfeld *et al*, [20] noted that 90%, 70%, and 60% respectively, were able to quantify the relative risk of breast cancer associated with family history, previous history of breast cancer, and smoking, respectively. The same authors found that over 70% of the surveyed women were able to identify painless breast lump, lump under the armpit and nipple discharge/bleeding as symptoms of breast cancer. It should however be noted that a much smaller proportion of these women were able to recognize other non-lump symptoms such as dimpling of the breast skin, inversion/pulling in of the nipple, and scaling/dry skin in the nipple region.

Our results indicate that education and employment in professional jobs significantly influenced knowledge of breast cancer. Women with education greater than High School and those employed in professional jobs such as nursing, teaching and sales had significantly higher knowledge scores compared with those employed in small businesses. Other demographic variables including age, marital status and religion were not significantly related to knowledge score. These results are in agreement with the findings of others but at variance with the report of others. Among a cross section of British women, Grunfeld *et al*, [20] found that older women demonstrated poorer knowledge of risk factors for breast cancer; they noted that this poorer knowledge was also apparent among women of lower social economic status (SES). Surveys in the US [21], and Australia [22] have demonstrated that older women have poorer knowledge of key risk factors for various cancers. It has been suggested that older women may attribute non-lump breast symptoms to the aging process, and therefore ignore these warning signs of breast cancer [20]. Furthermore, it has been argued that older adults, who may have a number of symptoms of other illnesses, should not be expected to seek help for symptoms that are not causing them any pain or that have little effect on their functioning [23].

Participants in our study had the right attitude towards breast cancer as majority indicated visiting the doctor for breast complaints. The use of screening methods was very low among our study subjects; only 34.9% practice BSE and only 9.1% had had CBE in the past year and none ever had a mammogram. Odusanya and Tayo [13] reported that 89% of Nurses in Lagos, Nigeria practiced BSE and 34.3% had CBE although majority of their study participants did not know the correct time or technique for carrying out the procedure. Available data indicates that majority of women in the screening age group in the developed countries undergo routine screening using all three methods including monthly BSE, annual CBE, and annual mammography [24,25]. In a survey of practice of BSE among black women in the US, Jacobs *et al*, [26] found that 89% of respondents indicated practicing BSE during the past year, 74% indicated having done so during the past six months, and 39% indicated performing self exam monthly. Similar percentage of US women reporting practice of BSE monthly or more often have been reported by other investigators [27].

Higher level of education and higher knowledge score were significant determinants of BSE practice in our study; age and other demographic variables were not significantly related to BSE practice. Similar to our findings, other investigators have reported that demographic characteristics such as higher levels of education and income, marital status, younger age, social support, knowledge and preventive attitudes, a history of breast diseases, a family history of breast cancer, having a regular physician, ethnic background and residence area are significant determinants of adherence to BSE practice [14,15,28].

The guidelines for breast cancer screening recommended by a consortium of American medical organizations including the American Cancer Society, stipulates that: between the ages of 40 and 49 years, women should undergo a CBE and mammography every year or two; women older than 50 years should have an annual CBE as well as a mammogram [29]. Mammography and CBE facilitate early detection and treatment of breast cancer, which is responsible for lower mortality rates [30]. In a screening setting, about 10% of breast cancers will only be detected by CBE [29].

The value of BSE is less established. While the findings of a clinical trial suggested that BSE results in no difference in risk of mortality from breast cancer, a review of case-control studies found that BSE might reduce this risk. Despite inconclusive evidence, it is thought that BSE makes women more "breast aware", which in turn may lead to earlier diagnosis of breast cancer [30]. The rationale behind extending BSE practice as a screening test is the fact that breast cancer is frequently detected by women themselves without any other symptoms. A meta-analysis of studies investigating the possible benefits of BSE has shown that regular practice increases the probability of detecting breast cancer at an early stage [14]. However, BSE is associated with other drawbacks including increased number of biopsies for benign breast lesions, [31,32] increased anxiety, and physician visits with consequent use of scarce health resources in addition to the distress, scarring and disfigurement that may be associated with breast biopsies.

Routine breast cancer screening is currently not being practiced in Nigeria. Even then, applying the recommended mammography screening guidelines in Nigeria will catch only a proportion of breast cancer cases as about 57% of breast cancer cases in Nigeria occur in women below the age of 50 years [3]. In addition, some other factors militate against routine breast cancer screening in Nigeria. The actual burden of breast cancer in the population is unknown due to lack of adequate cancer statistics. The age specific incidence of the disease needs to be established to make a case for routine screening of women of specific age groups. Women need to be "breast aware" to stimulate their interest in screening. Health care spending for chronic diseases in Nigeria is competing with several basic needs including provision of basic amenities and infrastructure, and control of several endemic childhood infections and parasitic infestations; any money invested in breast cancer screening must be justified by the benefits to the population. Given the non-availability of adequate data to justify mammography screening and the high cost and skilled expertise required for the procedure, current efforts at breast cancer screening in Nigeria must rely on a combination of BSE and CBE. Women can be taught the techniques of monthly BSE and nurses, midwives, and other healthcare providers can be trained to augment physicians in the performance of clinical breast examinations (CBE).

As previously indicated, the interviewer-administered questionnaire developed by the authors was the only instrument employed for recruitment of study participants. Although, this may limit comparability of our findings with that of other investigators, it is important to note that efforts were made to ensure some measure of validity by pre-testing the questionnaire on a convenient sample before commencement of the study.

## Conclusion

The results of this study have demonstrated the extremely low level of breast awareness among community-dwelling women in Nigeria. Until circumstances are ripe for routine mammography screening, emphasis should be on encouraging women to practice BSE and CBE. Health education programs should be targeted at women through various media including leaflets, television, and radio. In addition, health education should be channeled through women friendly agencies/organizations such as hospital antenatal and postnatal clinics, religious organizations, and Feminist organizations. Use of leaflets, although effective to some extent in literate societies, may be of limited value in the Nigerian population. Even in highly literate societies, there is evidence suggesting that leaflets produce only limited and short-lived changes in knowledge [20]. Furthermore, many health professionals believe leaflets are often not read by the target audience [33]. Television and radio appear to be better media to reach a wider audience but the benefits of these media may be limited in rural communities with limited access to these media. In the rural areas, it may be easier to reach a wide cross-section of women through organizations built around the pre-existing community institutional framework. Available data suggest that people prefer to learn about cancer-related issues from their doctors and health organizations. Within the hospitals, we suggest that breast awareness education be integrated into already existing health education programs. In addition, doctors should endeavor to educate women on "breast awareness" during regular physician office visits for other health issues. Non-governmental and other charitable organizations can also make significant contribution to "breast awareness" through sponsoring health talks, symposia and workshops targeted at relevant segments of the population.

## Competing interests

The author(s) declare that they have no competing interests.

## Authors' contributions

**MO**, **CB**, **UO**, **FO **participated in conceptualization, design of the study and preparation of manuscript;

**MO**, **CB**, **UO**, **FO **participated in data analysis and preparation of the manuscript.

All the authors read and approved the final manuscript.

## References

[B1] Parkin DM, Bray F, Ferlay J, Pisani P (2005). Global cancer statistics, 2002. CA Cancer J Clin.

[B2] Adebamowo CA, Ajayi OO (2000). Breast cancer in Nigeria. West Afr J Med.

[B3] Okobia MN, Osime U (2001). Clinicopathological study of carcinoma of the breast in Benin City. Afr J Reprod Health.

[B4] Anyanwu SN (2000). Breast cancer in eastern Nigeria: a ten year review. West Afr J Med.

[B5] Ihekwaba FN, Ihekwaba FN (1992). Breast cancer in Nigerian women
Breast cancer in Nigerian women. Br J Surg.

[B6] Neave LM, Mason BH, Kay RG (1990). Does delay in diagnosis of breast cancer affect survival?. Breast Cancer Res Treat.

[B7] Rossi S, Cinini C, Di Pietro C, Lombardi CP, Crucitti A, Bellantone R, Crucitti F (1990). Diagnostic delay in breast cancer: correlation with disease stage and prognosis. Tumori.

[B8] Machiavelli M, Leone B, Romero A, Perez J, Vallejo C, Bianco A, Rodriguez R, Estevez R, Chacon R, Dansky C (1989). Relation between delay and survival in 596 patients with breast cancer. Oncology.

[B9] Afzelius P, Zedeler K, Sommer H, Mouridsen HT, Blichert-Toft M (1994). Patient's and doctor's delay in primary breast cancer. Prognostic implications. Acta Oncol.

[B10] Richards MA, Westcombe AM, Love SB, Littlejohns P, Ramirez AJ (1999). Influence of delay on survival in patients with breast cancer: a systematic review. Lancet.

[B11] Peto R, Boreham J, Clarke M, Davies C, Beral V (2000). UK and USA breast cancer deaths down 25% in year 2000 at ages 20-69 years. Lancet.

[B12] Ramirez AJ, Westcombe AM, Burgess CC, Sutton S, Littlejohns P, Richards MA (1999). Factors predicting delayed presentation of symptomatic breast cancer: a systematic review. Lancet.

[B13] Odusanya OO, Tayo OO (2001). Breast cancer knowledge, attitudes and practice among nurses in Lagos, Nigeria. Acta Oncol.

[B14] Ferro S, Caroli A, Nanni O, Biggeri A, Gambi A (1992). A cross sectional survey on breast self examination practice, utilization of breast professional examination, mammography and associated factors in Romagna, Italy. Tumori.

[B15] Maxwell CJ, Bancej CM, Snider J (2001). Predictors of mammography use among Canadian women aged 50-69: findings from the 1996/97 National Population Health Survey. Cmaj.

[B16] Philip J, Harris WG, Flaherty C, Joslin CA (1986). Clinical measures to assess the practice and efficiency of breast self-examination. Cancer.

[B17] Smith EM, Francis AM, Polissar L (1980). The effect of breast self-exam practices and physician examinations on extent of disease at diagnosis. Prev Med.

[B18] Uche EE (1999). Cancer awareness among a Nigerian population. Trop Doct.

[B19] Odusanya OO (2001). Breast cancer: knowledge, attitudes, and practices of female schoolteachers in Lagos, Nigeria. Breast J.

[B20] Grunfeld EA, Ramirez AJ, Hunter MS, Richards MA (2002). Women's knowledge and beliefs regarding breast cancer. Br J Cancer.

[B21] Breslow RA, Sorkin JD, Frey CM, Kessler LG (1997). Americans' knowledge of cancer risk and survival. Prev Med.

[B22] Paul C, Barratt A, Redman S, Cockburn J, Lowe J (1999). Knowledge and perceptions about breast cancer incidence, fatality and risk among Australian women. Aust N Z J Public Health.

[B23] Ford G, Taylor R (1985). The elderly as underconsulters: a critical reappraisal. J R Coll Gen Pract.

[B24] Bjurstam N, Bjorneld L, Duffy SW, Smith TC, Cahlin E, Eriksson O, Hafstrom LO, Lingaas H, Mattsson J, Persson S, Rudenstam CM, Save-Soderbergh J (1997). The Gothenburg breast screening trial: first results on mortality, incidence, and mode of detection for women ages 39-49 years at randomization. Cancer.

[B25] Frisell J, Eklund G, Hellstrom L, Lidbrink E, Rutqvist LE, Somell A (1991). Randomized study of mammography screening--preliminary report on mortality in the Stockholm trial. Breast Cancer Res Treat.

[B26] Jacob TC, Penn NE, Brown M (1989). Breast self-examination: knowledge, attitudes, and performance among black women. J Natl Med Assoc.

[B27] Celentano DD, Holtzman D (1983). Breast self-examination competency: an analysis of self-reported practice and associated characteristics. Am J Public Health.

[B28] Maxwell AE, Bastani R, Warda US (2000). Demographic predictors of cancer screening among Filipino and Korean immigrants in the United States. Am J Prev Med.

[B29] Yucel A DBAMEHARHA (2005). Knowledge about breast cancer and mammography in breast cancer screening among women awaiting mammography. Turk J Med Sci.

[B30] Siahpush M, Singh GK (2002). Sociodemographic predictors of pap test receipt, currency and knowledge among Australian women. Prev Med.

[B31] Thomas DB, Gao DL, Ray RM, Wang WW, Allison CJ, Chen FL, Porter P, Hu YW, Zhao GL, Pan LD, Li W, Wu C, Coriaty Z, Evans I, Lin MG, Stalsberg H, Self SG (2002). Randomized trial of breast self-examination in Shanghai: final results. J Natl Cancer Inst.

[B32] Semiglazov VF, Manikhas AG, Moiseenko VM, Protsenko SA, Kharikova RS, Seleznev IK, Popova RT, Migmanova NS, Orlov AA, Barash NI, Ivanova OA, Ivanov VG (2003). [Results of a prospective randomized investigation [Russia (St.Petersburg)/WHO] to evaluate the significance of self-examination for the early detection of breast cancer]. Vopr Onkol.

[B33] Murphy S, Smith C (1993). Crutches, confetti or useful tools? Professionals' views on and use of health education leaflets. Health Educ Res.

